# Modulation of apolipoprotein E receptor-2 by ApoE4, amyloid β-peptide, reelin, and secreted amyloid precursor protein: a common point of impact in Alzheimer’s disease pathogenesis

**DOI:** 10.3389/fnmol.2026.1781541

**Published:** 2026-03-04

**Authors:** Steven W. Barger, Andréa M. Moerman-Herzog

**Affiliations:** 1 Department of Geriatrics, University of Arkansas for Medical Sciences, Little Rock, AR, United States; 2 Department of Neuroscience, University of Arkansas for Medical Sciences, Little Rock, AR, United States; 3 Geriatric Research Education and Clinical Center, Central Arkansas Veterans Healthcare System, Little Rock, AR, United States

**Keywords:** amyloid-beta, ApoER2, apolipoprotein E, calcium, lipoprotein receptor related protein 8, NMDA-receptor, reelin (RELN)

## Abstract

**Introduction:**

Apolipoprotein E (ApoE), reelin, and several other proteins bind ApoE-receptor 2 (apoER2), distinguished from other members of its receptor family by signal transduction which enhances the activity of N-methyl D-aspartate (NMDA) receptors. Evidence indicates that this signal transduction depends upon apoER2 forming dimers or other high-order clusters. It seems noteworthy therefore that protein products of major *APOE* gene variants differ in their numbers of cysteines capable of forming disulfide dimers, with the allele (ε4) associated with highest rates of Alzheimer’s disease (AD) possessing none. Thus, lower AD risk may be associated with the ability of ApoE to dimerize and thereby promote apoER2 dimerization and signaling.

**Methods:**

We examined calcium fluxes via the NMDA receptor in neurons derived from the NTera2 cell line in response to conditioned medium from human astrocytes differing in *APOE* genotype, recombinant ApoE proteins, reelin, amyloid β-peptide (Aβ) preparations differing in their aggregation states, and secreted amyloid precursor protein (sAPP). Signaling through apoER2 was inhibited by receptor-associated protein (RAP) or siRNA directed against apoER2.

**Results:**

Reelin, fibrillar Aβ, ApoE3, and conditioned medium from *APOE* ε3 astrocytes elevated calcium fluxes, and this phenomenon required apoER2. By contrast, ApoE4 and oligomeric Aβ antagonized activation. sAPP showed high-affinity binding to apoER2 and enhanced responses to reelin.

**Discussion:**

These findings suggest a comprehensive hypothesis for the pathogenesis of AD whereby the common factor in development of disease is antagonism of apoER2, likely to include agents that cannot promote the receptor’s dimerization yet competitively inhibit those ligands that can cause dimerization.

## 1 Introduction

The gene (*APOE*) for apolipoprotein E (ApoE) confers the largest genetic risk factor for late-onset Alzheimer’s disease (AD). Two single-nucleotide polymorphic sites in the coding region create three most common forms of the protein in humans: ApoE2, ApoE3, and ApoE4. Inheritance of one allele (ε4) encoding ApoE4 confers a 3.0–3.5 odds ratio for developing AD relative to individuals homozygous for the allele encoding ApoE3 (ε3;ε3) (Farrer et al., 1997). Homozygosity for the ε4 allele elevates the odds ratio to 11–15. This dose-dependency is a form of incomplete dominance and has been difficult to explain by simple loss-of-function or gain-of-function models of ApoE function. ApoE4 appears to be evolutionarily older than ApoE3; the latter differs from ApoE4 by a single amino acid substitution (Arg_112_ → Cys). ApoE2 differs from ApoE4 by two amino acids (Arg_112_ → Cys, Arg_158_ → Cys). Inheritance of a single ε2 allele encoding ApoE2 appears to confer protection against AD (OR = 0.76 vs. ε3;ε3) (Farrer et al., 1997). Thus, the risk for AD is inversely related to the number of cysteine residues in ApoE protein.

ApoE binds several members of the low-density lipoprotein (LDL) receptor family, including LDL-related protein (LRP) 1 and apoER2 (LRP8). Interactions between these receptors and ApoE-containing lipoprotein particles are important for the trafficking of cholesterol and triglycerides throughout plasma and interstitial spaces. However, two members of this receptor family—apoER2 and VLDL-R—participate in neurophysiology by facilitating synaptic plasticity, generally acting to enhance long-term potentiation (LTP) (Weeber et al., 2002; Zhuo et al., 2000), a memory-related phenomenon dependent upon the N-methyl D-aspartate (NMDA) class of glutamate receptors. VLDL-R and apoER2 are bound and activated not only by ApoE but also reelin, a protein critical for migration of neuroblasts during development and during adult neurogenesis (Rice and Curran, 2001). For at least some signaling responses, LRPs must be induced to multimerize, apparently to trigger phosphorylation of accessory proteins tethered to the cytoplasmic domains (Bacskai et al., 2000; Li et al., 2023; Strasser et al., 2004). This scheme is analogous to the activation of many receptors for cytokines, growth factors, and neurotrophins (Ferrari and Greene, 1996; Heldin and Ostman, 1996). In this regard, it seems noteworthy that ApoE3 exists as a disulfide dimer in plasma (Weisgraber and Shinto, 1991), cerebrospinal fluid (Rebeck et al., 1998; Yamauchi et al., 1999), and brain parenchyma (Elliott et al., 2010); whereas ApoE4 does not because it lacks cysteines.

Considerable evidence indicates that various forms of amyloid β-peptide (Aβ) are important in the pathogenesis of AD (Selkoe, 2008). It was recognized over two decades ago that long, fibrillar aggregates of Aβ exhibit a neurotoxicity *in vitro* that is correlated with their ability to exacerbate excitotoxicity through hyperactivation of NMDA-R (Koh et al., 1990; Mattson et al., 1992; Wu et al., 1995). However, more recent evidence suggests that soluble, oligomeric aggregates of Aβ are better correlated with cognitive decline and compatible physiological effects (Sakono and Zako, 2010). Unlike the larger fibrils, these small aggregates are consistently found to inhibit NMDA-R responses and LTP (Shankar et al., 2007; Walsh et al., 2002). Aβ is generated via coordinated cleavage of the amyloid precursor protein (APP), and other fragments of this protein have been characterized as neuroprotective. Specifically, secreted APP derived from α-secretase activity (sAPPα) protects hippocampal and neocortical neurons from excitotoxic, metabolic, and oxidative stresses (Jacobsen and Iverfeldt, 2009; Mattson, 1997).

It has been noted that many proteins connected to AD by genetics or biochemistry interact with members of the LRP family (Hyman et al., 2000; Rebeck et al., 1995). It is also notable that an important target of the signaling through these receptors is the NMDA-R, that this receptor is critical for the physiological substrates of memory, and that this receptor is significantly impacted by aggregates of Aβ. We hypothesized that the ligand-dependent dimerization of apoER2 (and possibly VLDL-R) is critical for memory-related actions of the NMDA-R and that monomeric ligands contribute to AD through antagonism of these LRP-family receptors. As an initial step in testing this hypothesis, we analyzed the extent to which Aβ’s neuromodulatory effects are dependent on apoER2, as well as the ability of ApoE3 and ApoE4 to activate apoER2. The results of these studies suggest that apoER2 is activated by fibrillar Aβ and ApoE3 but antagonized by oligomeric Aβ and ApoE4. Because APP has been shown to interact with reelin (Hoe et al., 2009) and also modulates the NMDA-R (Furukawa et al., 1996), we tested for its effects on the apoER2 pathway. Reelin actions on signal transduction events were augmented by preincubation with sAPPα. These findings support the hypothesis that AD develops as a result of inhibition of apoER2 (or related LRPs) by any of several antagonists that fail to dimerize the receptor—because the ligand is itself monomeric (ApoE4) or is too small to bridge the receptor dimer (oligomeric Aβ). Moreover, the beneficial effects of sAPPα may involve its enhancement of reelin activity.

## 2 Materials and methods

### 2.1 Materials

ApoE3 and ApoE4 were recombinant (baculovirus-expressed) proteins of human sequence obtained from Invitrogen (Carlsbad CA). A deletion mutant of sAPPα that lacks amino acids 1–303 (sAPPα_304–612_) was obtained from Sigma-Aldrich. Aβ_1–42_ was a recombinant preparation of Aβ_1–42_ generously provided as hexafluoroisopropanol (HFIP)-denatured, dessicated aliquots (rPeptide; Watkinsville GA). To prepare Aβ in a predominantly oligomeric state, denatured aliquots were dissolved at 2 mM in anhydrous dimethyl sulfoxide (DMSO) then diluted to 150 μM in ice-cold Minimal Essential Medium (MEM) and incubated for 24 h at 4°C; the resulting suspension was centrifuged for 10 min at 14,000 *g* at 4°C to remove potential fibrils. To prepare Aβ in a predominantly fibrillar state, the denatured aliquots were dissolved at 2 mM in DMSO then diluted to 150 μM in warm (37°C) 10 mM HCl and incubated for 24 h at 37°C. The preparations were characterized on 10–20% Tris-tricine gels, which showed a small amount of monomeric peptide in both preparations but scarcely any detectable oligomer in the fibrillar preparations. Similar physiological responses were obtained with aggregates prepared from HFIP-denatured preparations of synthetic Aβ_1–42_ obtained from Anaspec (Fremont CA).

Reelin was partially purified from serum-free medium (50/50 MEM/F12) conditioned by a HEK293 line stably transfected with reelin expression construct pCrl (D’Arcangelo et al., 1997) (courtesy of T. Curran; Children’s Hospital of Philadelphia Research Inst.). The conditioned medium (CM) was made 0.5 mM phenylmethylsulfonylfluoride (PMSF) and chilled to 4°C. A saturated solution of (NH_4_)_2_SO_4_ was then added gradually with continuous stirring to a concentration of 45%. After stirring ∼18 h at 4°C, the suspension was subjected to centrifugation at 20,000 *g* for 1 h at 4°C. Pellets were dissolved in Dulbecco’s phosphate-buffered saline (PBS), pH 7.4, 1 mM CaCl_2_, 0.5 mM MgSO_4_. This protein preparation was dialyzed against the above solution for 1 h at 4°C to remove excess ammonium sulfate. Glycerol was then added to 25% before storage at −80°C. Reelin concentrations were approximated by densitometric comparison of the major bands to those produced by known quantities of bovine serum albumin in Coomassie-stained SDS-PAGE.

Recombinant human sAPPα was purified from serum-free culture medium (50/50 MEM/F12) conditioned by a HEK293 line stably transfected with an APP695 cDNA into which a stop codon was inserted after the sixteenth amino acid in the Aβ domain. Conditioned medium from these lines was passed through a DE-52 anion exchange column, which was then washed with phosphate-buffered saline (PBS) and step-eluted with PBS containing 0.75 M NaCl. The sAPP-containing fractions were pooled and loaded onto a fast-preparative liquid chromatography (FPLC) system equipped with a Hi-Trap heparin-Sepharose column (GE Life Sciences), which was then washed with PBS and eluted with a linear gradient of PBS to PBS + 1M NaCl. The sAPP-containing fractions from the heparin column were pooled and resolved by FPLC using a MonoQ anion-exchange column (GE Life Sciences), which was washed with Buffer A (20 mM triethanolamine-HCl, 100 mM NaCl, pH 7.4) and then eluted with a linear gradient of Buffer A to Buffer B (20 mM triethanolamine-HCl, 1 M NaCl, pH 7.4). The sAPP-containing fractions were pooled and dialyzed against Buffer C (124 mM NaCl, 26 mM NaHCO_3_, 3 mM KCl, 2 mM CaCl_2_ 1.4 mM MgCl_2_ 1.25 KH_2_PO_4_, pH 7.4) before storage at −80°C. Binding studies were conducted with sAPPα that had been labeled with an AlexFluor^®^ 568 protein labeling kit (Molecular Probes/Invitrogen) according to manufacturer’s directions.

### 2.2 Cell cultures

The NTera2 cell line (American Type Culture Collection) was maintained in MEM supplemented to 10% with fetal bovine serum (FBS). The cells were differentiated to neuron-like cells (hNT) by plating into bacteriological dishes in the presence of 10 μM all-*trans* retinoic acid for at least 14 days (with fresh medium changes every 2–3 days). The neurospheres that form under these conditions were trypsinized and plated as a dissociated cell suspension in MEM with 5% FBS on glass-bottomed 35-mm dishes that had been coated with 100 μg/cm^2^ polyethyleneimine. The following day, the medium was supplemented to 4 μM cytosine D-arabinofuranoside (araC) 20 μM uridine to suppress the numbers of nonneuronal cells. HEK293 cells were maintained in MEM/10% FBS.

Human astrocytes were obtained from tissue of the superior temporal gyrus at autopsy. The tissue was trypsinized, dissociated, and grown in MEM/10% FBS for approximately 1 month. The cells were then suspended in a medium containing 10% DMSO and frozen in liquid nitrogen until use. To generate conditioned media, frozen vials were thawed rapidly and seeded into MEM/20% FBS. After 2 days, the FBS concentration was reduced to 10% and the cultures were expanded. When confluent, the cultures were washed in serum-free MEM and placed in a 50:50 mixture of MEM and F12 medium supplemented with 10 nM sodium selenite and 50 μM ethanolamine. After 3 days, the conditioned medium was collected and concentrated on Centricon filters by 30-fold. The concentrated medium was divided into aliquots and stored at −80°C.

Primary cultures of rat cortical neurons were established in 60-mm dishes as described previously (Mao et al., 2009). On day 6 *in vitro*, half the culture medium (Neurobasal/B27; Invitrogen) was replaced with fresh medium, and the cultures were exposed to stimuli on day 8 *in vitro*.

The High Five insect cell line (Invitrogen) was maintained in Express Five SFM (Invitrogen). Suspensions of these cells were transfected with an apoER2 expression vector (below) using Cellfectin (Invitrogen). The cells were then plated into opaque black 96-well plates for fluorescence binding assays (below).

### 2.3 Measurements of intracellular free calcium concentration ([Ca^2+^]_*i*_)

The [Ca^2+^]_*i*_ of hNT cells was monitored by dual-wavelength ratiometric measurements of fura-2 as described previously (Barger and Basile, 2001), except that the imaging buffer was supplemented with 5 μM glycine. Each tracing represents the mean of ≥32 cells from three separate cultures. Unless otherwise noted, statistical analysis was performed on the time point providing the peak of the mean [Ca^2+^]_*i*_, integrated with two readings before and after it. For pairwise comparisons, the means of these integrated values were subjected to an unpaired *t-*test. For analysis of experiments containing more than two conditions, ANOVA was followed by Bonferroni *post-hoc* analysis. *P* ≤ 0.05 was considered to be significant.

### 2.4 RNAi treatments

The siRNA treatments were performed essentially as described (Barger et al., 2008) using either control dsRNA (Santa Cruz cat # sc-37007) or a pool of dsRNA sequences directed against human apoER2 (Santa Cruz cat # sc-40097).

### 2.5 apoER2 binding assays

For testing the binding of sAPPα to apoER2, a cDNA encoding a variant of the receptor containing Exon 19 was inserted into the pIB/V5-His plasmid (Invitrogen). High Five cells were transfected in suspension with 10 μg of the parent plasmid (“mock”) or pIB-apoER2(long) and 6 μL Cellfectin per million cells. After 4 h, the cells were plated into a white opaque 96-well plate. After 2 days, the medium was replaced with ice-cold SFM and various concentrations of sAPPα tagged with Alexa Fluor^®^ 568; some wells were preincubated 10 min with unlabeled sAPPα (“+comp.”) or reelin (“+reelin”). After 2 h at 4°C, the wells were gently washed twice with ice-cold PBS, then lysed with PBS containing 0.5% SDS. Lysates were measured on a SpectraMax M2 fluorometric plate reader with excitation at 565 nm and emission at 615 nm. Values were interpolated into a standard curve generated with the tagged sAPPα in lysis buffer.

### 2.6 Dab1 phosphorylation assay

Primary cultures of cortical neurons were exposed to stimuli for 15 min, washed once with ice-cold PBS, then lysed in ice-cold RIPA buffer. Lysates were subjected to centrifugation at 12,000 g for 10 min at 4°C, and the supernatants were assayed for protein concentration by BCA assay (Pierce). An aliquot of each was removed as an “input” reference, and aliquots containing equal amounts of protein were precleared with a slurry of protein-A/G agarose beads (Pierce/Thermo Scientific) then incubated overnight at 4°C with rabbit anti-Dab1 (Rockland). The antibody-lysate mixtures were mixed by rotation with protein-A/G slurry for 2 h at 4°C, then the beads were collected by centrifugation at 12,000 *g* at 4°C. The supernatants were removed for storage, and the pellets were washed with ice-cold RIPA buffer then collected again by centrifugation. The pellets were heated to 100°C in Laemmli sample buffer, and the supernatants from this step were resolved by SDS-PAGE (8%). The gels were transferred to nitrocellulose membranes and subjected to western blot analysis (similar to that for binding assays above) with mouse anti-phosphotyrosine (1:1,000; 4G10, Millipore).

## 3 Results

### 3.1 ApoE3 stimulates and ApoE4 inhibits apoER2

**FIGURE 1:**
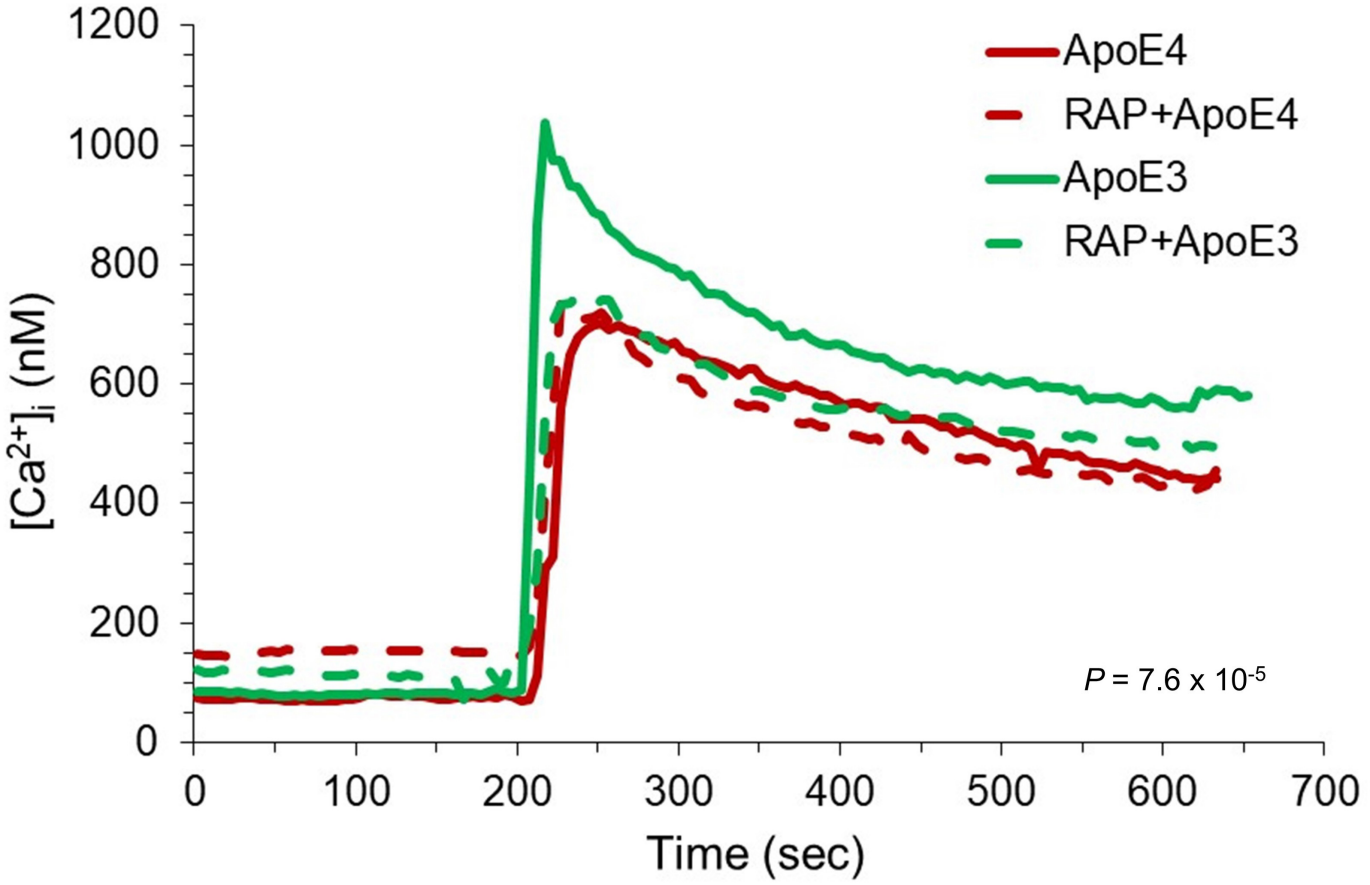
Elevation of neuronal [Ca^2+^]_*i*_ by human astrocyte-conditioned medium. Human astrocytes homozygous for *APOE* ε3 (green) or ε4 (red) were cultured, and conditioned medium (CM) was obtained and concentrated as described in Materials and Methods. CM was applied to hNT neurons during microfluorometric monitoring of [Ca^2+^]_*i*_; CM from each cell type was also tested after a 3-min pretreatment with 200 nM receptor-associated protein (RAP). Initial concentration of the CM combined with the dilution into the imaging bath resulted in a net dilution of 1:17 in the imaging bath buffer. The solid tracings represent the mean [Ca^2+^]_*i*_ in cells treated with CM alone; the dashed tracings represent the mean in cells treated with CM following RAP pretreatment. *P* = 7.6 × 10^–5^ for the peak [Ca^2+^]_*i*_ in ApoE3 CM vs. all other groups (1-way ANOVA and Bonferroni *post hoc* analysis).

At least one study found that recombinant ApoE can act as an antagonist of apoER2, inhibiting the ability of reelin to stimulate phosphorylation of the accessory protein Dab1; no difference was noted between E3 and E4 versions of the protein (D’Arcangelo et al., 1999). However, a peptide comprising a tandem repeat of ApoE’s receptor-binding domain activated Dab1 phosphorylation (Hoe et al., 2005), suggesting that dimeric ApoE (e.g., ApoE3) might also act as an agonist. As our initial test of the effects of native ApoE on this system, we monitored NMDA-R-dependent changes in [Ca^2+^]_i_ in response to conditioned medium (CM) from human astrocytes obtained from individuals who were homozygous for the ε3 or ε4 allele. CM from cultures of three separate ε3;ε3 individuals were assessed independently and compared to CM from cultures of two separate ε4;ε4 individuals. The medium samples were applied to hNT neurons (differentiated from the human NTera2 cell line) while [Ca^2+^]_i_ was monitored by fura-2 ratiometric fluorescence imaging (Figure 1). CM from ε4;ε4 cultures caused a three- to four-fold increase in [Ca^2+^]_i_ in hNT cells, but the response to CM from ε3;ε3 cultures was approximately twice this magnitude.

**FIGURE 2:**
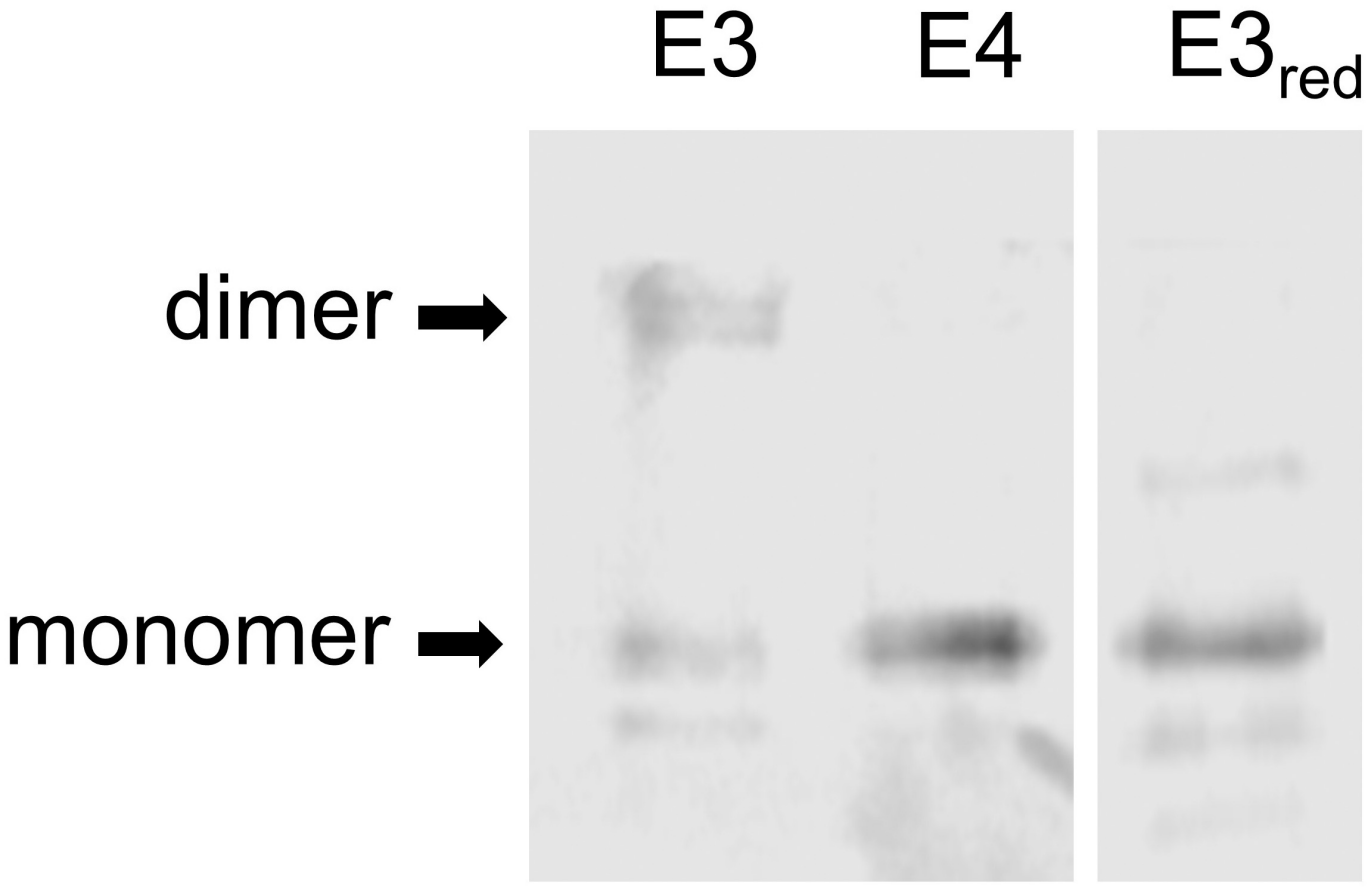
Dimeric nature of recombinant ApoE3. The commercial preparation of rApoE3 used in these studies was resolved on SDS-PAGE in unreduced conditions (lane 1) or after reduction with dithiothreitol (lane 3). These were compared to the rApoE4 run in an unreduced state (lane 2).

**FIGURE 3:**
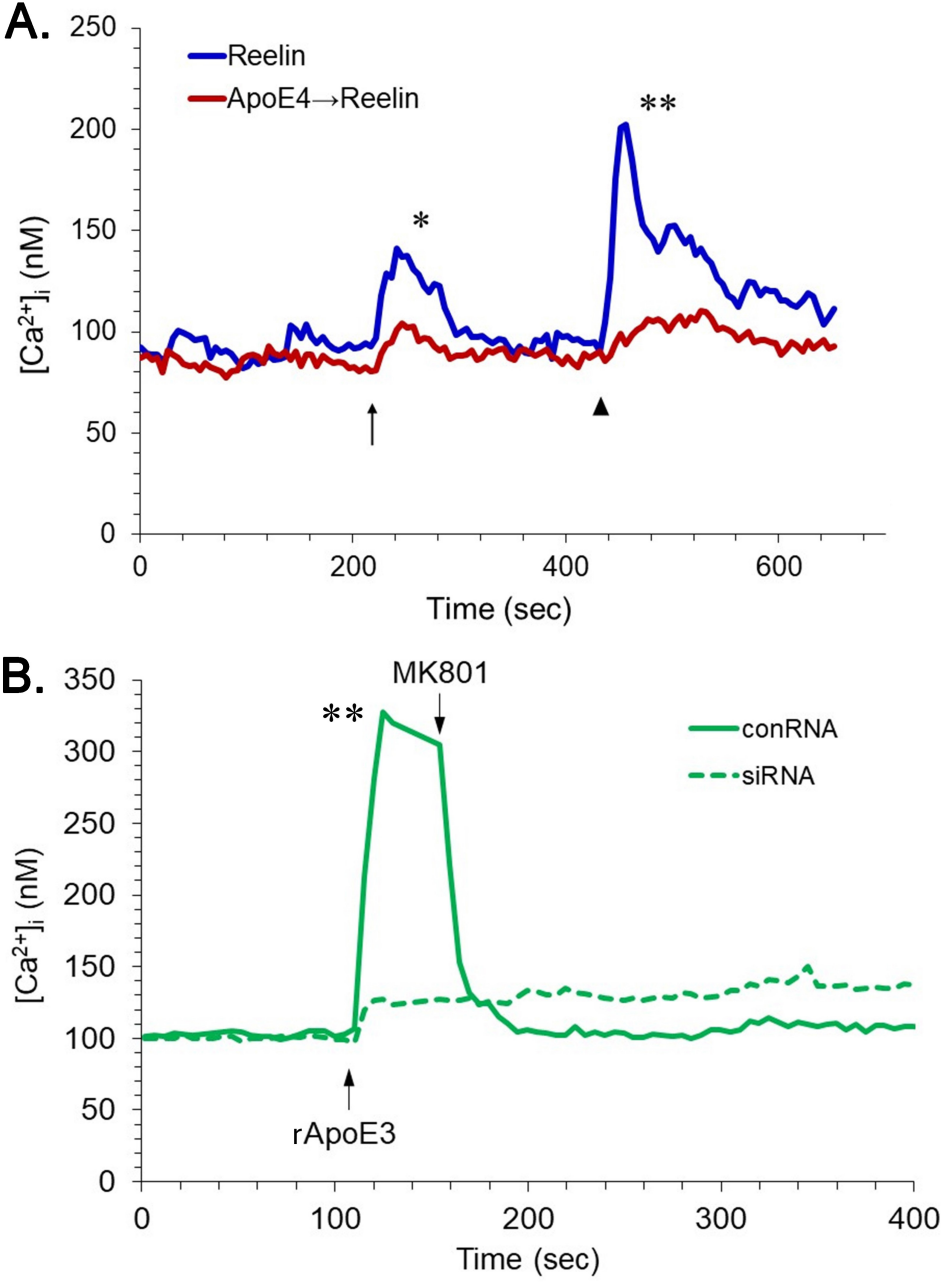
Divergent effects of rApoE3 and rApoE4 on neuronal [Ca^2+^]_*i*_. The hNT neurons were monitored for [Ca^2+^]_*i*_ during application of reelin and/or rApoE. **(A)** The small arrow indicates the time at which reelin was applied at 10 nM; the arrowhead indicates the application of reelin at 50 nM. The blue tracing represents [Ca^2+^]_*i*_ in cells treated with reelin alone; the red tracing reflects [Ca^2+^]_*i*_ in cells treated with rApoE4 (22 nM) prior to the initiation of [Ca^2+^]_*i*_ monitoring (*P<0.05, reelin alone vs. ApoE4+reelin at 280 sec; **P<0.01, reelin alone vs. ApoE4+reelin at 640 sec). **(B)** The hNT neurons were treated either with siRNA directed against apoER2 (dashed line) or with control RNA (solid line). After 48 h, the cells were then monitored for [Ca^2+^]_*i*_ during application of rApoE3 (22 nM). At the time indicated, NMDA-R antagonist MK801 (50 μM) was added to the conRNA cultures (**P<0.01, cRNA vs. siRNA at 140 sec).

While the results of these conditioned-medium experiments suggested that ApoE3 might promote a larger response than ApoE4, they are subject to the caveat that individual humans exhibit considerable genetic variation, and we utilized cultures from a limited number of individuals. It is also likely that the entire elevation of [Ca^2+^]_i_ was not triggered by ApoE alone, as the CM certainly contained additional cellular factors, perhaps including glutamate or other neurotransmitters. To circumvent these issues, we tested recombinant ApoE (rApoE) in similar assays. We have previously noted physiological effects on neurons in response to rApoE3, effects that differed qualitatively from data obtained with rApoE4 (Barger et al., 2008; Barger and Harmon, 1997). SDS-PAGE separation showed the rApoE3 to contain a substantial amount of dimer in non-reducing conditions, whereas rApoE4 was entirely monomeric, as was rApoE3 in the presence of dithiothreitol (Figure 2). When rApoE4 was applied at 22 nM to hNT cells, there was no elevation in [Ca^2+^]_i_. Indeed, it inhibited responses to reelin (Figure 3A). By contrast, rApoE3 evoked a rapid and substantial increase in [Ca^2+^]_i_ (Figure 3B). This effect was dependent upon NMDA-R, as it was sensitive to the antagonist MK801. Presumbably, hNT cells engage in sufficient basal neurotransmission (Hill et al., 2012) that enhancement of the NMDA-R component of this activity can be observed without exogenous agonists.

To ascertain that the elevation of [Ca^2+^]_i_ by rApoE3 involved apoER2, expression levels of the receptor were reduced by siRNA. A commercially provided pool of siRNA sequences was tested at two concentrations, and a 50 nM application was found to deplete levels of apoER2 in hNT cells to ∼25% of controls (Supplementary Figure 1). Though the inhibition of apoER2 expression by siRNA was incomplete, it was likely to be sufficient if propagation of the receptor’s signal depends on homodimerization. Such a scenario would produce responses exhibiting second-order kinetics and therefore decreasing exponentially with diminution of the receptor’s steady-state levels, reaching an approximate inhibition of 94%. Indeed, the ability of rApoE3 to elevate [Ca^2+^]_i_ was greatly diminished by apoER2 siRNA (Figure 3B). (hNT cells not treated with RNA responded similarly to those treated with control RNA; data not shown).

### 3.2 Aβ fibrils and oligomers differentially impact apoER2

We were intrigued by studies suggesting that aggregated Aβ accentuated responses of the NMDA-R, especially in light of later reports that Aβ oligomers inhibited the same (Shankar et al., 2007). We hypothesized that large fibrils of Aβ might be capable of fostering dimerization of apoER2 through direct binding, as Aβ has been shown to bind LRP1 (Deane et al., 2004; Laporte et al., 2004). Oligomeric Aβ, on the other hand, might bind to apoER2 without the capacity to span a receptor dimer, potentially creating a competitive inhibition. This hypothesis was made more compelling after the demonstration that Aβ oligomers inhibit reelin’s actions (Durakoglugil et al., 2009).

**FIGURE 4:**
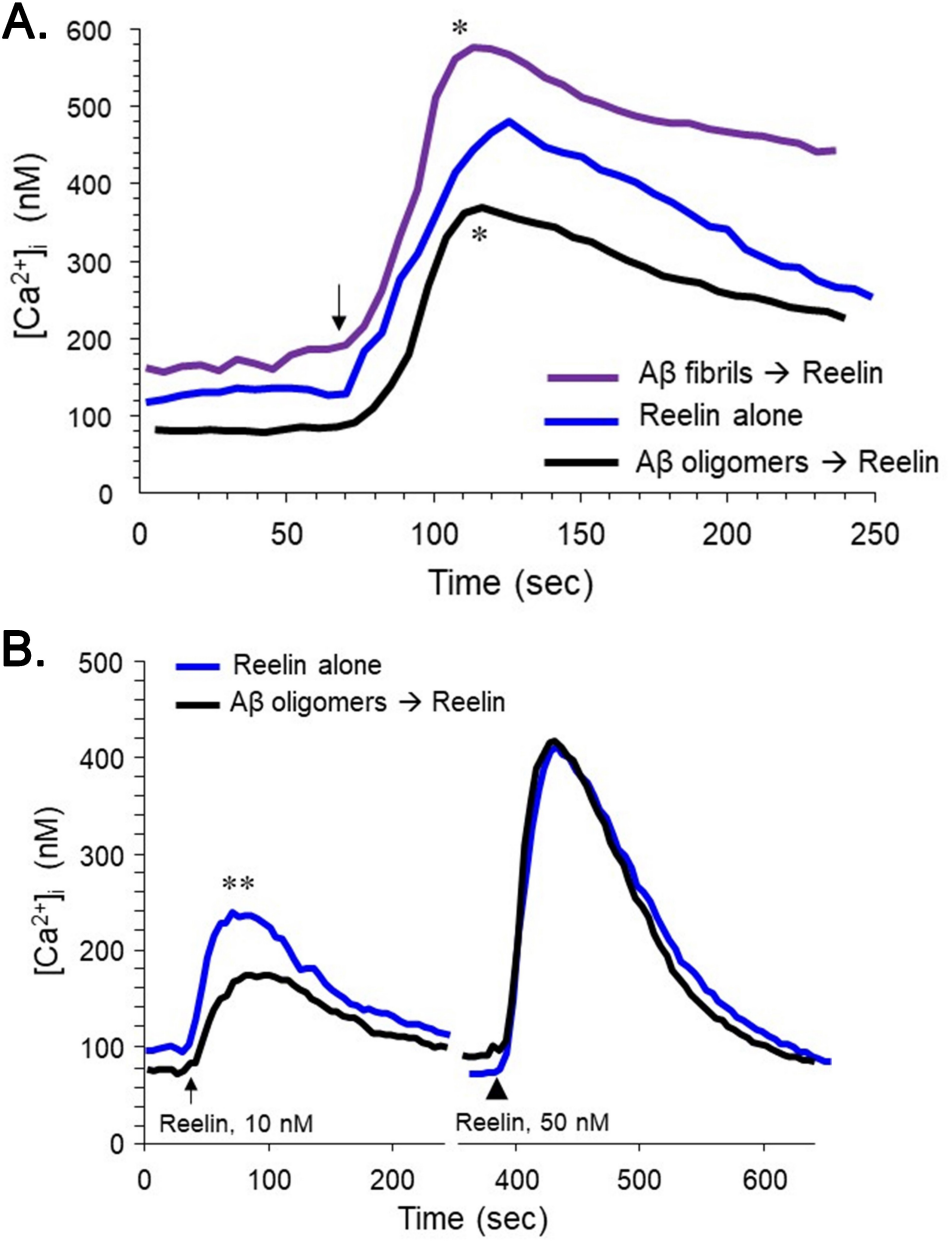
Divergent effects of fibrillar and oligomeric Aβ on neuronal [Ca^2+^]_i_. The hNT neurons were monitored for [Ca^2+^]_i_ during application of reelin with or without a 2-min pretreatment with Aβ_1–42_ (5 μM). **(A)** The Aβ was prepared under conditions to generate fibrils or oligomers; reelin was applied at 10 nM. Each trace represents 43–51 cells in three cultures (*P < 0.05, vs. reelin alone at peak [Ca^2+^]_i_). **(B)** Two concentrations of reelin (10 and 50 nM) were applied with or without a pretreatment of oligomeric Aβ. Each trace represents 57–64 cells in three cultures [**P < 0.01, vs. Aβ+reelin at peak (Ca^2+^)_*i*_].

**FIGURE 5:**
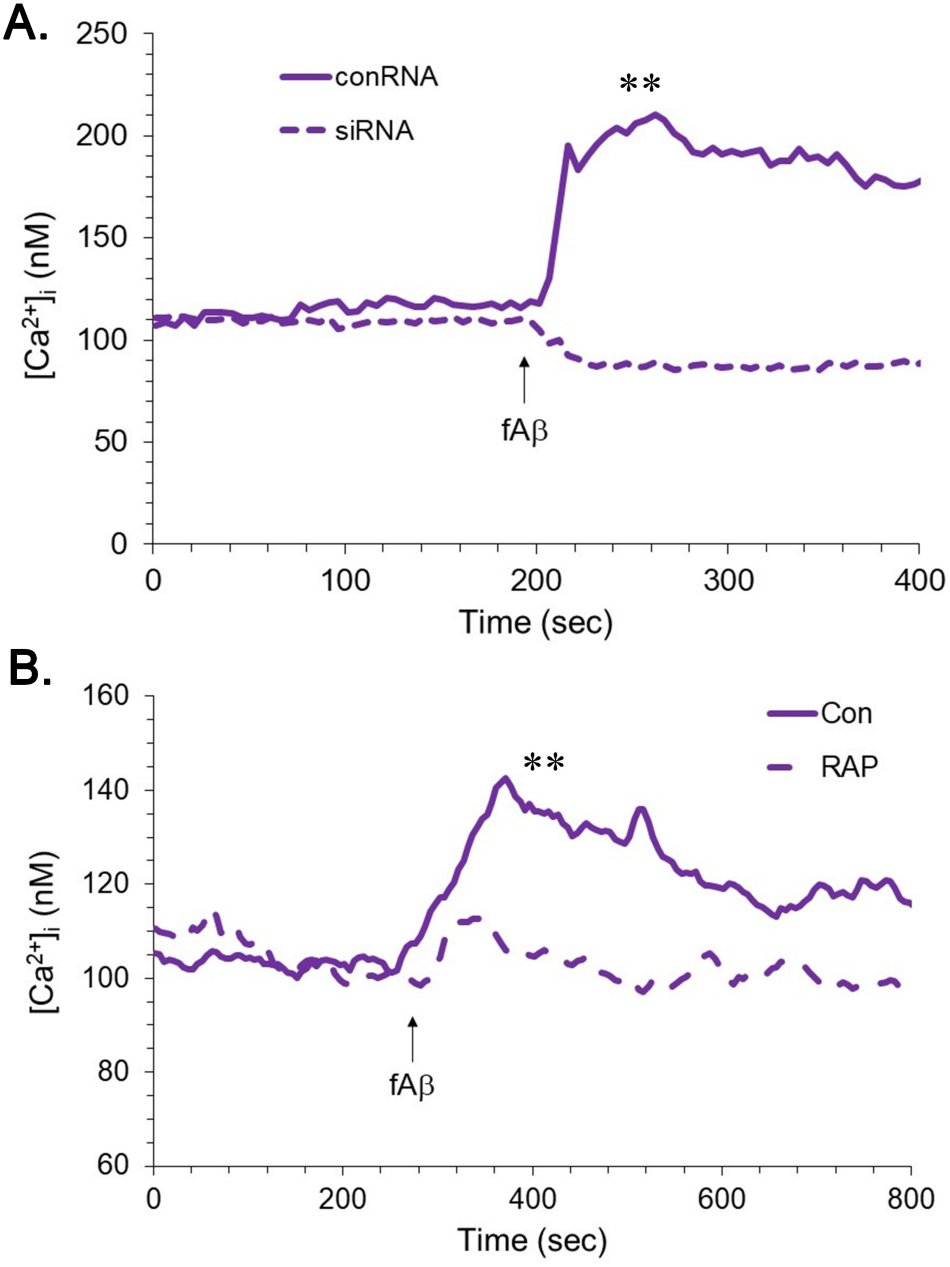
Elevation of [Ca^2+^]_i_ by fibrillar Aβ is dependent upon apoER2. The hNT neurons were monitored for [Ca^2+^]_i_ during application of a fibrillar preparation of Aβ_1–42_. **(A)** The hNT neurons were treated either with siRNA directed against apoER2 or with control RNA; 48 h later, 5 μM Aβ was applied at 200 s. Each trace represents 47–54 cells in 3 cultures (**P < 0.01 vs. siRNA). **(B)** Some hNT cells were pretreated for 3 min with 200 nM RAP and compared to naïve cells in their responses to 1 μM Aβ. Each trace represents 32–34 cells in 3 cultures (**P < 0.01 vs. RAP+Aβ).

Potential interactions of Aβ with apoER2 were tested through functional assays as for ApoE. Application of a preparation of Aβ_1–42_ that was predominantly fibrillar triggered an elevation of [Ca^2+^]_i_ (Figures 4A, 5). As with the response to ApoE, the elevation in [Ca^2+^]_i_ by fibrillar Aβ was reduced by > 95% with MK801 (not shown). Pretreatment of hNT cells with oligomeric Aβ_1–42_ was associated with a lower basal [Ca^2+^]_i_ and blunted responses to reelin (Figure 4A). This inhibition of reelin responses appeared to be competitive, as it could be overcome with an increased concentration of reelin (Figure 4B).

To more incisively test the role of apoER2 in the responses to Aβ, two approaches were taken. First, hNT cells were treated with siRNA directed against apoER2. These conditions converted the effect of fibrillar Aβ from an elevation of [Ca^2+^]_i_ to a diminution (Figure 5A), reminiscent of the acute effect of sAPPα (Barger et al., 1995; Mattson et al., 1993). We also tested the effect of fibrillar Aβ in the presence of receptor-associated protein (RAP), an LRP-family antagonist. At 200 nM, RAP significantly inhibited the response to 1 μM fibrillar Aβ (Figure 5B). Some evidence indicates that Aβ can elevate [Ca^2+^]_i_ through the activation of metabotropic receptors, specifically mGluR5 (Renner et al., 2010). We tested this mechanism in our preparations of hNT cells and found no inhibition by 10 μM SIB1757, an mGluR5 antagonist (Supplementary Figure 2).

### 3.3 Secreted APPα enhances reelin activity

**FIGURE 6:**
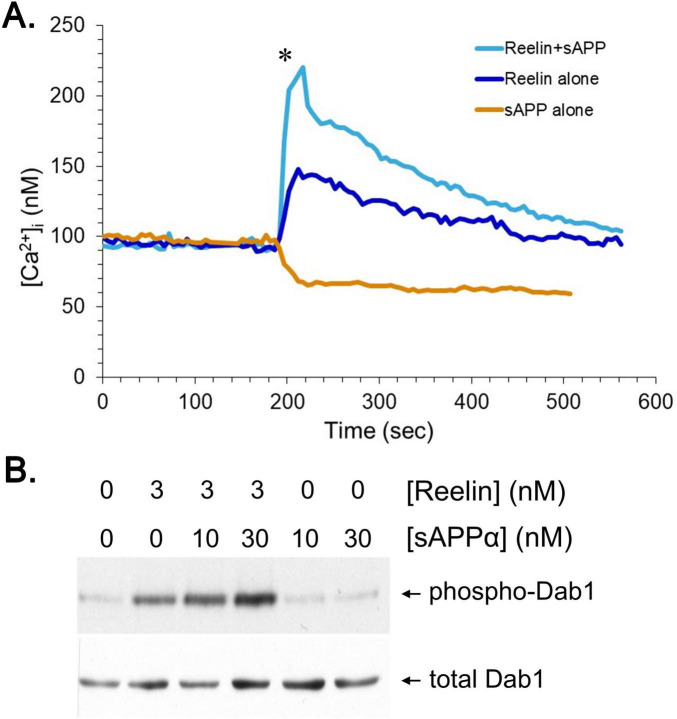
Enhancement of reelin responses by sAPPα. **(A)** The hNT neurons were monitored for [Ca^2+^]_i_. At the time indicated by the arrow, cultures were treated with either reelin alone, sAPP alone, or a combination of reelin and sAPP preincubated for 30 min at 1.5 μM. Each protein was 30 nM in the final imaging buffer. Each trace represents 39–53 cells from three cultures. The peak values obtained after reelin/sAPP treatment were significantly higher than those after reelin alone (*P < 0.02). **(B)** Primary cortical neurons were treated for 15 min with reelin (3 nM), sAPP (10 or 30 nM), or a combination of reelin and sAPP that had been preincubated together for 30 min. Cultures were lysed and subjected to immunoprecipitation with anti-Dab1, followed by western blot analysis with anti-phosphotyrosine. Aliquots of the lysate prior to immunoprecipitation were subjected to western-blot analysis with the anti-Dab1 antibody to visualize total Dab1 levels.

Reelin and APP interact physically, and a decrease in APP expression diminishes reelin’s effects on neurite outgrowth (Hoe et al., 2009). We considered the possibility that the beneficial roles of APP and its secreted fragments—namely, sAPPα—might involve effects on reelin activity. To test this hypothesis, reelin and sAPPα were combined in solution to allow the formation of heteromeric complexes between the two proteins. These mixtures were compared to reelin or sAPPα alone in assays of neuronal [Ca^2+^]_i_ and Dab1 phosphorylation. Preincubation with sAPPα for 30 min significantly enhanced reelin’s ability to elevate [Ca^2+^]_i_ in hNT cells (Figure 6A). This effect appeared to involve physical interactions between sAPPα and reelin because a deletion construct of sAPPα that is incapable of interacting with reelin (sAPPα_304–612_) did not enhance reelin’s effects on [Ca^2+^]_i_ (data not shown). As reported previously (Mattson et al., 1993), sAPPα alone caused a decrease in resting [Ca^2+^]_i_. Reelin-evoked phosphorylation of Dab1 was also enhanced by preincubation with sAPPα (Figure 6B).

**FIGURE 7:**
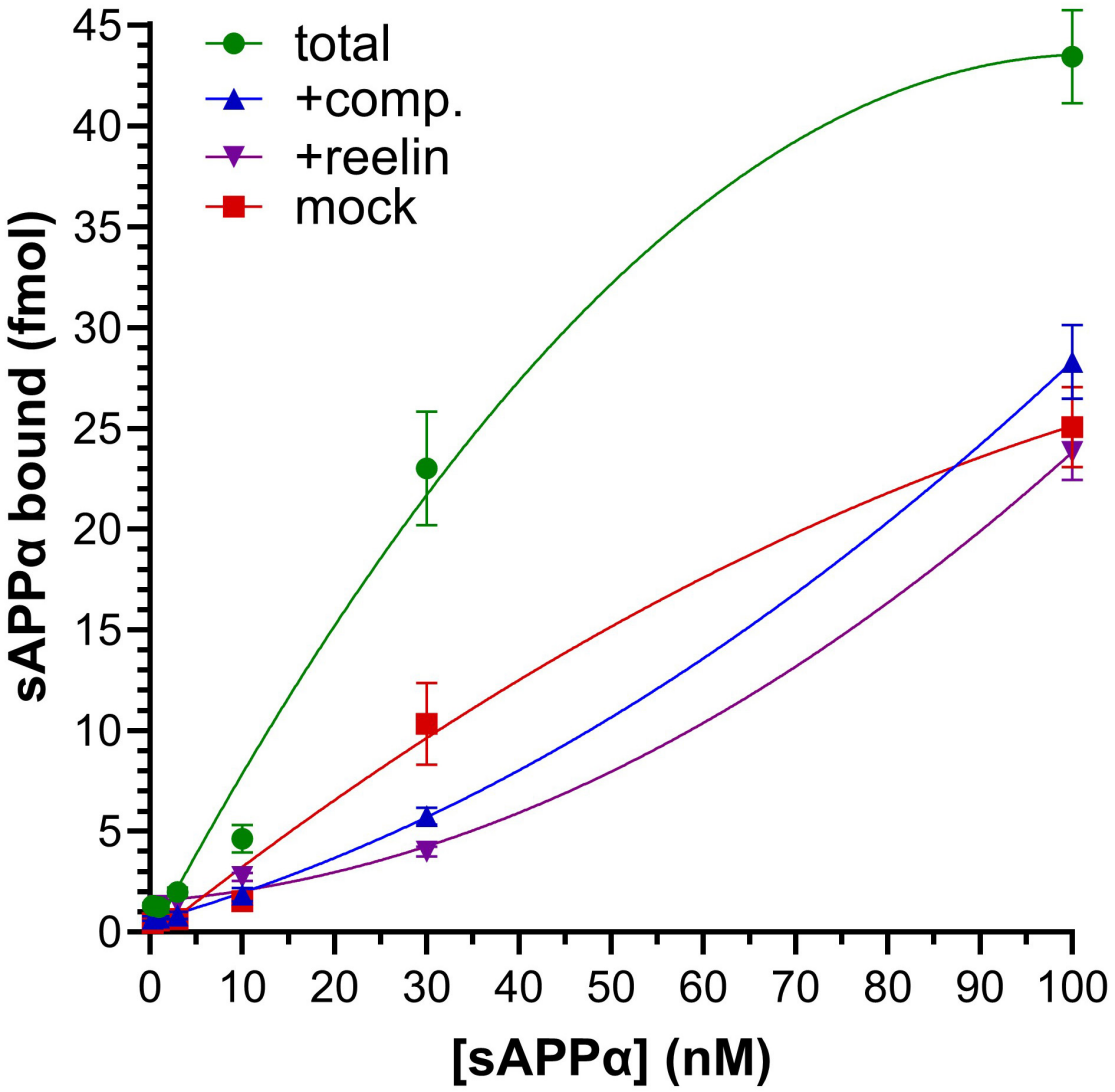
sAPPα binds apoER2. An insect cell line was transfected with an apoER2 expression vector and exposed to 0.3–100 nM fluorescently labeled sAPPα alone or after application of 10 μM unlabled sAPPα (“+comp.”) or reelin (“+reelin”). Fluorescent sAPPα was also tested in cultures transfected with the empty parent vector (“mock”). Values represent mean ± SEM of quadruplicate cultures. The “total” curve is significantly different from all other curves: *P* < 0.0001 (ANOVA and Bonferroni *post hoc*).

It is possible that sAPPα interactions with reelin alter conformation of the latter to enhance interactions with its receptors. Alternatively, sAPPα might bind apoER2 itself and participate in multimerization of the receptors as part of a sAPPα-reelin heterodimer or other, higher-order multimers. The splice variants of APP containing a Kunitz protease inhibitor (KPI) domain have been shown to bind to other lipoprotein receptors, namely LRP1 (Kounnas et al., 1995). However, the functional effect of sAPPα on reelin bioactivity above was achieved with the variant of sAPPα lacking the KPI (i.e., that derived from APP695). We generated fluorescently tagged sAPPα (AF568-sAPPα) to assay binding to apoER2, with the latter expressed in an insect cell line through transient transfection. AF568-sAPPα showed saturable binding in cells transfected with apoER2 that was considerably higher than that observed in cells transfected with empty vector (“mock”) (Figure 7). Inclusion of 100x unlabeled sAPPα (“+comp.”) reduced binding of AF568-sAPPα to levels closer to those observed in mock-transfected cells. Subtracting the values obtained in the presence of unlabeled competitor from the total binding values yielded specific binding consistent with a K_D_ of ∼19 nM. Binding of AF568-sAPPα to apoER2-transfected cells was also competed away with reelin.

## 4 Discussion

A prominent hypothesis for AD pathogenesis revolves around demonstrations that ligation and/or trafficking of LRP1 impacts the processing of APP (Bu et al., 2006). This idea suggests that LRP1 metabolism is but one of several paths to excessive production of Aβ, itself viewed as a final common mediator for AD pathogenesis. Here we consider a hypothesis that places the LRP-family receptors in a more central role; namely, that antagonism of their signal transduction is the final common mediator for development of dementia. In this scheme, accumulation of Aβ—particularly, oligomers—is but one of several paths to this antagonism. We found that oligomeric preparations of Aβ_1–42_ inhibited the elevation of [Ca^2+^]_i_ by reelin, whereas fibrillar preparations elevated [Ca^2+^]_i_ on their own. The latter appeared to involve apoER2 binding, as it was inhibited by siRNA directed against this receptor and by RAP. Similar inhibitions of the [Ca^2+^]_i_ elevation triggered by ApoE3 suggest for the first time that ApoE dimers can be agonists of the signal-transducing LRPs, perhaps explaining the biological effects attributed to tandem-repeat peptides based on ApoE’s receptor-binding domain (below). ApoE4, which has no potential for disulfide-linked dimerization, acted as an antagonist. Fundamentally, this appears similar to inhibitory effects attributed to ApoE4 in previous studies (e.g., Chen et al., 2010); however, the rapid time frame of the effects we observed indicate an acute competition rather than chronic receptor down-regulation. Together, these results suggest that disulfide dimers of ApoE3 and longer fibrils of Aβ_1–42_ are capable of assembling dimers (or higher order clusters) of apoER2, activating the signal transduction cascade and thereby mimicking or supplementing the activity of reelin. Monomeric ApoE4 and smaller aggregates of Aβ appear to be incapable of assembling apoER2 and thus bind as competitive antagonists.

It has been noted that most ligands of LRP1 are associated with AD either genetically, neuropathologically, or both (Hyman et al., 2000; Rebeck et al., 1995). However, most of these ligands have also been shown to bind the signaling members of the LRP family, apoER2 and VLDL-R. Moreover, sporadic AD does not appear to require overproduction of Aβ (Ray et al., 1998). It is possible that some forms of dementia arise via apoER2 antagonism without elevations in Aβ deposition. Nevertheless, the hypothesis developed here predicts that all antagonists present would make quantitative contributions to disease risk and progression. Thus, factors that alter Aβ production or oligomerization, factors that alter the availability of reelin or ApoE3 dimers, and factors that alter apoER2 downstream signaling would still modify disease onset and progression in ε4 carriers, creating a potential for the sort of variability observed in age-related aspects of human cognition.

Although we were not able to evaluate the biological activity of variants other than ApoE3 and -4, our model makes predictions about the genetic influence of *APOE* ε2. The protective effect of ApoE2 could result from its second cysteine residue simply providing greater opportunity and stability for covalent ApoE dimers (perhaps including ApoE2-ApoE3 dimers). Notably, our hypothesis would predict that ApoE2 would not provide much protection in the context of the ε2;ε4 genotype because it would not be capable of dimerizing with the product of the other diploid copy: ApoE4. It might confer a slightly lower AD risk than the ε3;ε4 genotype merely as a result of its greater incidence of homoallelic dimerization; i.e., ApoE2-ApoE2 dimers being more favorable than ApoE3-ApoE3 dimers. This prediction of the dimerization hypothesis is born out by meta-analysis of *APOE* odds ratios (ORs): ε2;ε4 has an OR of 2.6 (versus the indexed genotype: ε3;ε3), which is not significantly different from the OR of 3.2 seen in ε3;ε4 (with an overlap of 1.2 in the 95% confidence interval) (Farrer et al., 1997). If ApoE2 was protective via some mechanism independent of dimerization, it would likely also be independent of the other diploid copy—i.e., unaffected by the absence of Cys_112_ in the ApoE4 sequence. Thus, it would be expected to reduce the OR of ε4^+^ individuals as significantly as it does for ε3^+^ individuals. The fact that ε2 significantly lowers the rate of AD in ε3^+^ individuals but not ε4^+^ individuals suggests that ApoE2 functionally interacts with the product of the other diploid copy; one explanation would be physical dimerization of ApoE2 with ApoE3 but not ApoE4, a scenario consistent with the cysteine content of the various isoforms.

The overall hypothesis of apoER2 antagonism is consistent with spatial-memory deficits reported for ε4 carriers at young ages (Acevedo et al., 2010). Still, the hypothesis must be able to accommodate the age dependency of AD genetics. The effect of age may be explained by a quantitative antagonism of apoER2, which would likely present as phenotypically distinct from a qualitative loss of apoER2 signaling, as would occur with genetic ablation, for instance. Specifically, the antagonism is envisioned as being mild enough to produce dementia only in the aged brain, when other stresses have reduced the functional capacity of neural networks to a point where the impact of apoER2 antagonism becomes important. These other stresses may include a decline in neurogenesis (below). It is also possible that the summed antagonism reaches a threshold of clinical dementia only after an age-dependent increase in soluble Aβ (Pop et al., 2010).

We have used NMDA-R-mediated elevations of [Ca^2+^]_i_ as the primary index of apoER2 activation. This would be consistent with the hypothesis that the pathogenesis and clinical presentation of AD and other dementias result from an interference with plasticity at existing synapses. To wit, declines in synaptic activity appear to promote AD-related neuropathology (Tampellini et al., 2010). However, it is possible that other consequences of interrupting the apoER2 signaling cascade are more important for the sequence of events that eventually contribute to AD. Indeed, the connections of reelin and apoER2 to genesis and migration of adult neuroprogenitor cells (Haas and Frotscher, 2010), along with emerging evidence for epileptiform activity in AD (Palop and Mucke, 2009), suggest that antagonism of apoER2 causes disease by generating ectopic distribution of newborn hippocampal neurons. Data from experimental models of epilepsy indicate that the loss of reelin-producing interneurons removes a signal necessary for proper localization of neuroprogenitors in the dentate gyrus (Gong et al., 2007; Haas et al., 2002; Haas and Frotscher, 2010; Heinrich et al., 2006). Experimental disruption of reelin signaling creates such abnormalities in neuron location and fosters the development of seizure activity in mice (Gong et al., 2007; Heinrich et al., 2006). It is possible that the decline of neurogenesis that naturally occurs during aging (Jessberger and Gage, 2008) conspires with apoER2 antagonists to disrupt the proper cellular organization of the dentate gyrus and, perhaps through disinhibition, foster hyperstimulation of cornus ammonis 1. Mechanistically, this would likely involve loss of inhibitory GABAergic influence (Dudek and Sutula, 2007). The results of experimental disruption of reelin signaling provide empirical evidence that loss of an excitatory gain (normally provided by apoER2 agonists) can be converted through this sort of “sign change” into an excitotoxic form of cell death in the hippocampus, perhaps analogous to the neurotoxicity of NMDA-R antagonists studied extensively by Olney (Olney et al., 1991). ApoE4 targeted-replacement mice have an age-dependent reduction in hilar GABAergic interneurons (Li et al., 2009). This may explain the increased tendency toward epileptiform activity in ε4 carriers and their first-degree relatives (Palop and Mucke, 2009).

The ability of sAPPα to enhance reelin’s bioactivity is consistent with protective roles proposed for APP (Jacobsen and Iverfeldt, 2009; Mattson, 1997). We previously reported a decline in APP expression in spatial proximity to amyloid plaques in AD and suggested that this phenomenon might contribute to dysfunction and/or neuronal cell death (Barger et al., 2008). Most inferences about sAPP neuroprotection rely on cell culture models, where enhancement of neuronal survival tends to be correlated with decreases in [Ca^2+^]_i_. Here, in contrast, the agents hypothesized to protect against the development of dementia elevated [Ca^2+^]_i_. It is important to note that neurotoxicity can arise from neurophysiological activity being either too high or too low, and in cell culture the effect of a given agent depends largely on where the culture conditions place the cells with regard to their “calcium set-point” (Johnson et al., 1992). *In vivo*, neurons are substantially protected from potential excitotoxicity via robust uptake and amination of glutamate by astrocytes (Anderson and Swanson, 2000). For these reasons, bath application of excitatory amino acids to neurons *in vitro* can produce results that are quite different from excessive synaptic activity *in vivo*; excitotoxicity *in vitro* may not always be relevant to *in vivo* situations. Being pushed above their optimal activity set-point is something to which CNS neurons appear more vulnerable in culture, making the depression of [Ca^2+^]_i_ uniquely beneficial in that setting. Neuroprotective effects have also been reported for Aβ under certain conditions (Atwood et al., 2003; Giuffrida et al., 2009), conceivably related to the inhibition of [Ca^2+^]_i_ elevations by oligomers reported here. In the intact brain, diminution of apoER2 signaling by sAPP may be a more relevant contributor to symptomology than is excitotoxicity. The other side of this coin is represented by a peptide bearing a tandem repeat of ApoE’s receptor-binding domain, which has been shown to elevate [Ca^2+^]_i_ (Wang and Gruenstein, 1997) and produce NMDA-R-dependent excitotoxicity in culture (Qiu et al., 2003). Presumably, this tandem peptide mimics dimeric ApoE (E2 and E3) and thereby provides yet another discrepancy between *in vitro* excitotoxicity and *in vivo* dementia. It is also possible that antagonism of apoER2 results in diminutions in the activity of existing synapses, which then promotes AD-related pathology (Tampellini et al., 2010).

ApoE has been shown to act as an antagonist at apoER2 in past studies (Chen et al., 2010; D’Arcangelo et al., 1999). In one case, no difference was noted between rApoE3 and rApoE4 (D’Arcangelo et al., 1999). It is possible that the particular preparation of rApoE3 utilized did not have the opportunity to form disulfide-linked dimers, as the elicitation of apoER2-dependent effects we observed for rApoE3 was associated with the appearance of a dimer on non-reducing SDS-PAGE. It was recently reported that ApoE4 could act as an antagonist by ligand-induced receptor internalization; ApoE4 was found to slow the rate at which apoER2 was recycled and returned to the cell surface (Chen et al., 2010). This mechanism does not appear to be responsible for the phenomenon we observed because the effects were very rapid; even in cases in which ApoE was applied prior to reelin, the former was present for < 2 min in advance. According to Chen et al. (2010), there would be no difference between the effects of ApoE3 and ApoE4 on receptor internalization/recycling at this time point.

While ApoE4 may diminish steady-state levels of apoER2 on the cell surface, we propose that ApoE4, RAP, and other monomeric ligands also act as competitive antagonists that simply interfere with the binding of agonists. It appears that activation of apoER2 is consistent with a ligand’s ability to form aggregates large enough to bridge two subunits of the receptor and thereby stablize their dimerization. For reasonably large proteins such as reelin and ApoE, this may require only a dimeric form of the ligand. To wit, the single cysteine capable of forming a disulfide bridge in ApoE3 is critical to this hypothesis and to its ramifications for the mechanistic explanation of AD genetics. For Aβ, dimers and other low-order aggregates may not be large enough to span the steric gap between receptor subunits whereas fibrils could. It is also possible that the structure of a soluble Aβ oligomer is qualitatively distinct from the fibrillar form such that key constraints on receptor binding/dimerization are not satisfied. If binding of Aβ to apoER2 requires the N-terminus of the peptide, for instance, this domain might be exposed at only one end of a “head-to-tail” arrangement, thus creating an aggregate that is monovalent regarding the N-terminal domain.

Reelin was previously shown to antagonize the synaptic effects of oligomeric Aβ (Durakoglugil et al., 2009). That study also documented the converse: Aβ oligomers inhibited reelin’s stimulation of NMDA-R subunit phosphorylation. However, this effect was not attributed to direct interactions between Aβ and apoER2. Nevertheless, those results are consistent with our data showing that Aβ oligomers can inhibit reelin-evoked [Ca^2+^]_i_ elevation.

The proinflammatory aspects of AD pathology may also involve interactions with LRP-family receptors. It is possible that ApoE receptor signaling contributes to an anti-inflammatory state, an idea supported by the finding that a tandem repeat of the ApoE receptor-binding domain exerts an inhibitory effect on microglial activation (Pocivavsek et al., 2009). A similar peptide consisting of only a single copy of the receptor-binding domain was ineffective in this regard (G.W. Rebeck, “Effects of apoE receptors on APP trafficking and processing,” at *ApoE, ApoE Receptors and Neurodegeneration*; St. Louis MO, 7 June 2010), suggesting that receptor dimerization (and thus, activation) is necessary for the effect. ApoE3 has been reported to have anti-inflammatory activity (Laskowitz et al., 1998; Lombardi et al., 1998), and ApoE4 has proinflammatory effects (Colton et al., 2002a; Colton et al., 2002b; Lombardi et al., 1998; Rodriguez et al., 2014). These phenomena could reflect their differential effects on ApoE receptors.

The hypothesis suggested by the current data presents challenges for the development of therapies. All of the natural agonists of apoER2 are large, multimeric proteins that would be difficult to deliver directly into the hippocampus or other CNS locations. Intracerebroventricular cannulation and administration of reelin has been effective in modifying the neurophysiology of mice, but this approach is not ideal for long-term human therapy. The mimetic peptide representing a tandem repeat of ApoE’s receptor-binding domain might be delivered to the CNS more easily and efficiently, but peptide-based therapies still present challenges. Nevertheless, the small size of the effective peptide does offer hope that the steric requirements of spanning the receptor dimer are not beyond the reach of a small-molecule ligand that might be produced through rational drug design. Alternatively, small-molecule therapy might be able to bypass the receptor itself and activate appropriate signal transduction cascades. As proof of principle, it may be possible to advance this line of investigation through *in vivo* studies in which the levels of apoER2 (and VLDLR) are manipulated via RNAi, loss- or gain-of-function mutations, or transgenesis.

## Data Availability

The raw data supporting the conclusions of this article will be made available by the authors, without undue reservation.

## Supplementary material

Supplementary Figure S1 Depletion of apoER2 expression with siRNA. The hNT neurons were transfected as described in Materials and Methods with control RNA or siRNA directed against apoER2 at 10 or 50 nM. After 48 h, lysates were prepared, and equal protein amounts were subjected to western blot analysis for apoER2 **(A)**. The graph **(B)** shows the mean of densitometric quantification in duplicate experiments ± S.D.

Supplementary Figure S2 Elevation of [Ca^2+^]_i_ by fibrillar Aβ is independent of mGluR5. The hNT neurons were monitored for [Ca^2+^]_i_ during the application of 5 μM fibrillar Aβ_1–42_ (arrow). Sister cultures were subjected to the same treatment in the presence of 100 μM SIB1757 (arrowhead). Each trace represents the mean of 35–41 cells in three cultures.
