# Supplementary figures and images for: Modulation of apolipoprotein E receptor-2 by ApoE4, amyloid β-peptide, reelin, and secreted amyloid precursor protein: a common point of impact in Alzheimer’s disease pathogenesis
Source: Front Mol Neurosci. 2026 Mar 4;19:1781541. doi: 10.3389/fnmol.2026.1781541 (PMC12995784; doi:10.3389/fnmol.2026.1781541)

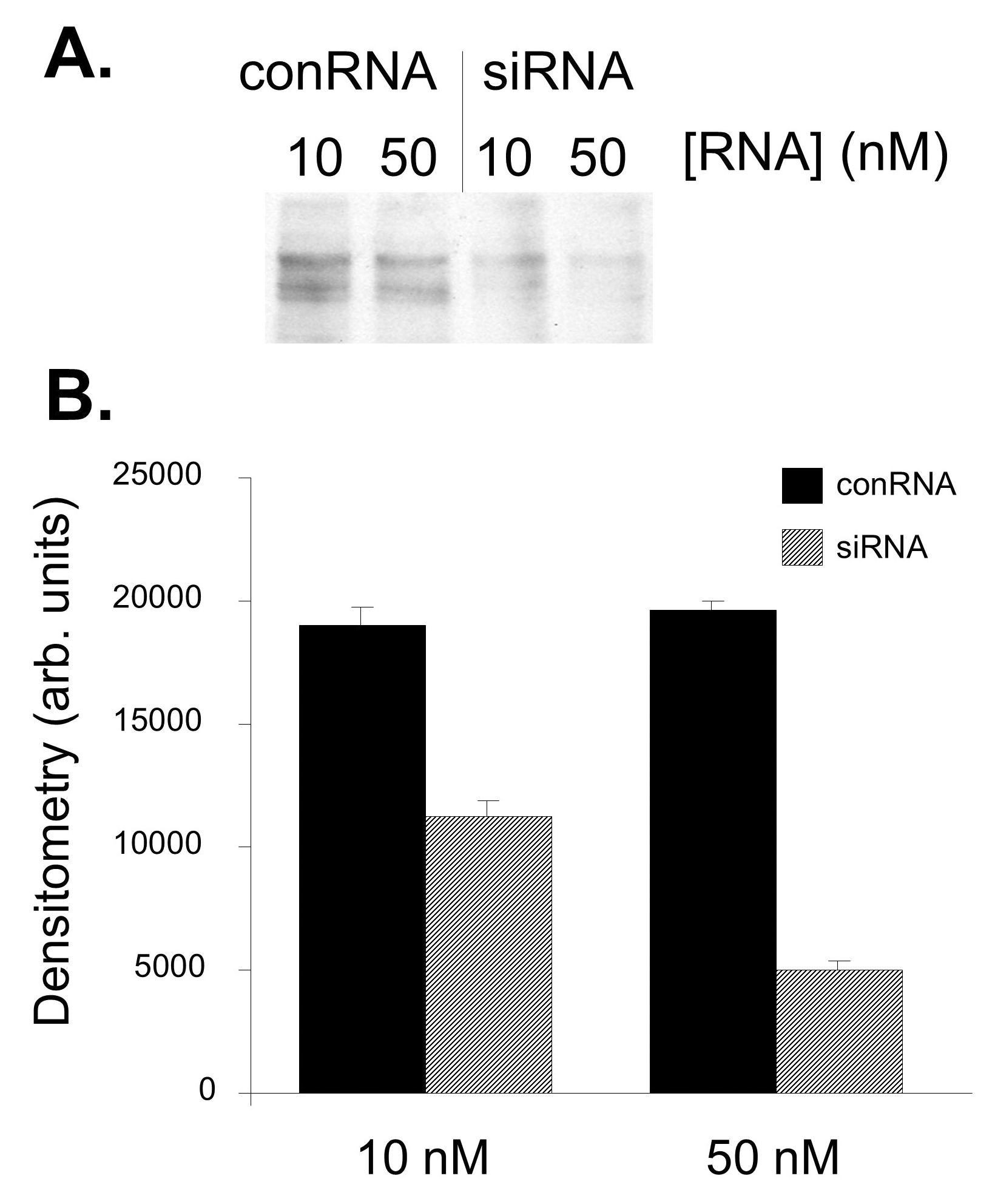

Supplement: Supplementary Figure S1 — Depletion of apoER2 expression with siRNA. The hNT neurons were transfected as described in Materials and Methods with control RNA or siRNA directed against apoER2 at 10 or 50 nM. After 48 h, lysates were prepared, and equal protein amounts were subjected to western blot analysis for apoER2 (A). The graph (B) shows the mean of densitometric quantification in duplicate experiments ± S.D. [file Image_1.jpeg]

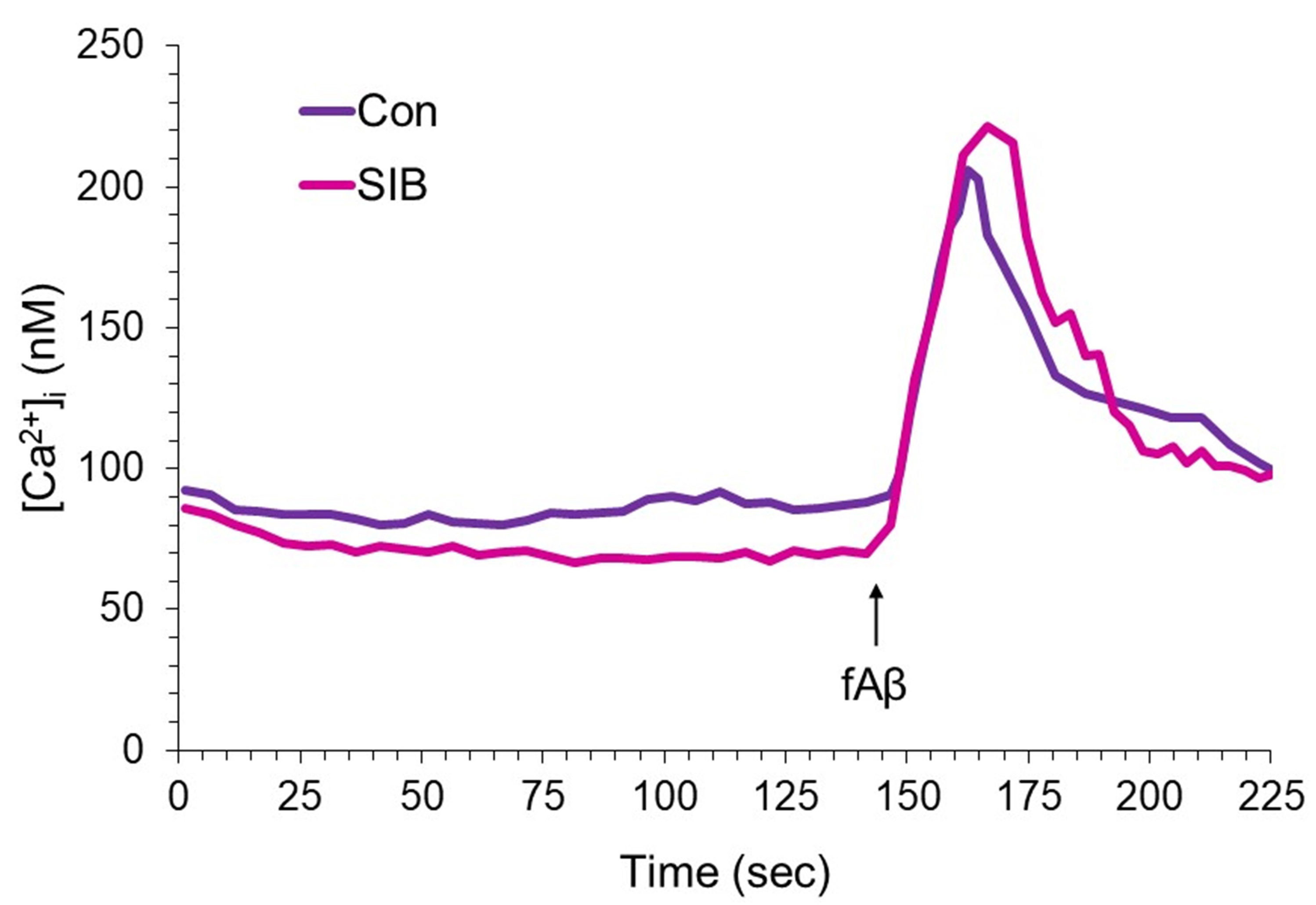

Supplement: Supplementary Figure S2 — Elevation of [Ca2+]i by fibrillar Aβ is independent of mGluR5. The hNT neurons were monitored for [Ca2+]i during the application of 5 μM fibrillar Aβ1–42 (arrow). Sister cultures were subjected to the same treatment in the presence of 100 μM SIB1757 (arrowhead). Each trace represents the mean of 35–41 cells in three cultures. [file Image_2.jpeg]
